# Mr. Cunningham, on Scruple Doses of Calomel

**Published:** 1818-05

**Authors:** James Cunningham

**Affiliations:** Surgeon. His Majesty's Ship Rochefort


To Dr. JAMES JOHNSON.

My dear Sir,

PURSUANT to the promise I made you, some time ago, to furnish you with a few cases illustrative of the advantages of exhibiting the submuriate of mercury, in scruple doses, in the cure of syphilis; and during the last twelve years, having employed the medicine in that proportion in a considerable number of cases, of almost every form of the disease (as my different Journals sent to the Transport Office exhibit in detail); I have chosen one or two, in various stages of the disorder, as a specimen, which I shall narrate as concisely as possible, though it may be, at the same time, necessary to be somewhat circumstantial.

Concluding from my great success in the cure of dysentery, by scruple doses of calomel, on board the Sceptre in India, as already promulgated in your excellent work on the Influence of Tropical Climates, &c. that the advantage of administering the medicine in that proportion might be extended to other diseases, I determined on trying its effects in syphilis, as soon as an opportunity offered.

In September, 1805, being fellow passenger to England, on board His Majesty's ship Weymouth, with Captain D. R. N. he consulted me for a chancre on the glans penis, that had resisted the remedies employed at Madras hospital for a long time. I suggested to him the propriety of attacking the disease by scruple doses of calomel, to which he readily consented; he was therefore purged, and in the evening he swallowed a bolus, containing a scruple of the submur. hydrarg. (sine opio), which neither produced purging nor any other intestinal uneasiness worth noticing: the ulcer was touched with the argent. nitrat. No alteration was made in his diet or beverage, there being a preliminary stipulation to that effect! The following evening he took another dose with the same effect, only the next morning his mouth was sensibly affected, and the mercurial fœtor was distinguishable in his breath, and the sore had assumed a more healthy aspect. His mouth was sorer the following day, and for some time afterwards; the chancre began to heal kindly. A week hence he took another scruple, which kept up a slight soreness in his mouth for a fortnight, when he took another. The sore healed progressively; twenty grains more were administered, and in little more than a month the sore was perfectly healed, and continued permanently cured. During the above period, he daily partook of the mess-fare, and drank his pint of Madeira at dinner regularly!

In 1806, being surgeon of H. M. S. Gibraltar, in the channel, various opportunities offered for giving the remedy further trial; I shall therefore pass over a tedious case, in September, of obstinate paraphymosis with ulcers, connected with a painful stricture in the urethra, accompanied with inflammation and induration externally. Likewise another, at the same period, in which the patient complained of an elastic swelling of the left testis, apparently a deposition of water within the tunica vaginalis, with a considerable enlargement of the spermatic cord, in which the scruple doses of calomel were exceedingly advantageous. In the latter case, they excited absorption in a surprising degree; for by that means, with confinement to his hammock, and the parts being covered with adhesive plaster, the patient, in the course of four months, recovered, so as to become fit for his duty, without any operation, and the testis afterwards returned to its natural size. Besides some other good cases, which would be too tedious and monotonous to narrate and detail, an admirable instance, in which, through the stimulus of the medicine, the energy of the absorbents, in removing venereal excrescences, was most conspicuous and remarkable.

About four months previous to the 1st of March, 1807, John Bennett joined the Gibraltar from Plymouth hospital, where he had been for the cure of lues venerea. At this period he complained of numerous clusters of excrescences about the scrotum, perineum, and anus, some of which were as large as an ordinary sized nutmeg, accompanied with an exudation of a very disagreeable fœtor. He complained also of a distressing pain in his back, which he attributed to a fall he received from the mast-head of some ship. He was put on the sick-list and on low diet, took a purge in the morning, and at six o'clock in the evening he swallowed four grains of opium; (in the temperature of the Channel, I did not usually, in syphilitic affections, administer scruple doses of calomel without preceding the dose by a proportion of opium;) and two hours afterwards he took a bolus, containing a scruple of the submur. hydrarg.

2d. Bowels slightly moved by the medicine; parts washed with an astringent lotion. *Vesp.* Opium and calomel repeated as before.

3d. Was a good deal griped last night, and purged towards the morning; gums inflamed and swelled, and his breath has the mercurial fœtor: a rawness which existed around the glans penis is already healing.

4th. Mouth sore; no griping or purging; pain of back gone; excrescences evidently begin to diminish in size. Hab. mist. diaphoret. anodyn. ter in die.

5th. Some ptyalism; mouth sore. Mist. contin.

6th. Ptyalism and soreness of mouth greatly subsided; excrescences disappearing. *Vesp.* Opium and calomel repeated.

7th. No particular griping or purging; the largest excrescence is scarcely half its original size, and many of the smaller ones have altogether disappeared.

8th. Mouth not very sore; no ptyalism; diaphoretic mixture as formerly. *Vesp.* Opium and calomel repeated.

9th. No particular uneasiness produced by the medicines; has had two or three loose stools this morning; excrescences daily diminishing. Mist. diaphoret. continuetur ut antea.

12th. Mouth scarcely sore; very little of the eruption now remains Ut antea, opium et calomel repetantur.

13th. No griping, but he has had six or seven stools this morning; mouth little affected. He declares he never enjoyed better health. All the excrescences, with a tetter which covered the inside of his thighs, except the base of the two largest on the lower part of the scrotum, have entirely disappeared, and they too are almost removed.

17th. Little or no soreness of mouth; opium and calomel repeated.

18th. Several loose stools, and was a good deal griped. Diaphoretic mixture continued.

20th. Omit the diaphoretic mixture, et sumat. mist. cort. Peruvian. ℥ij ter.

26th. Excrescences entirely disappeared; the mark of the base of the two largest is now only perceptible, and he has returned to his duty in excellent health.

May 15th, 1807. Edward Fagan applied for several chancres about the prepuce, frænum, and on the penis externally, as far down as the scrotum. He was put on low diet, took a purge, and in the evening a scruple of calomel, preceded by three grains of opium about an hour before.

16th. No purging from the medicines; sores washed with a solution of the sulphate of copper. Opium and calomel repeated in the evening.

17th. Mouth becoming sore; breath fœtid; ulcers clean, and discover a healing disposition.

18th. Sores treated as before; opium and calomel repeated in the evening.

19th. No particular effect from the medicine, except the system being fully impregnated with the mercury. Med. omit.

20th. Recovering rapidly; wash continued.

27th. Ulcers perfectly healed, and continued so.

May 16th, 1807. Robert Crawford complained of a chancre on the glans penis. Put on low diet, had a purging draught, and in the evening hyd. submur. ℈j, preceded by three grains of opium.

17th. Medicine produced no effect on his bowels; ulcer washed with a detergent lotion; calomel and opium repeated in the evening.

18th. Sore clean and contracting fast; mercurial fœtor very strong in his breath.

19th. Wash only continued.

24th. Chancre perfectly cured.

June 4th, 1807. Charles Corbett complained of two chancres, one on the prepuce, the other on the glans penis, accompanied with a bubo in his right groin. Put on low diet, had a cathartic, and, after its operation, three grains of opium, followed by a scruple of calomel.

5th. Was neither griped nor purged; chancres touched with the argent. nitrat.; no application to the bubo. Opium and calomel repeated in the evening.

6th. Medicines produced sickness at stomach, and towards the morning he had several loose stools.

7th. Bowels still relaxed. Opium and calomel repeated in the evening.

8th. Chancres much better, and the bubo is disappearing fast. Mouth and breath affected.

9th. Bowels quite easy. Opium and calomel repeated in the evening.

10th. No particular effect from the medicines.

12th. Ulcers healing. Opium and calomel repeated.

13th. No griping or purging; bubo entirely gone.

15th. Opium et calomel repetat.

16th. No purging; mouth very sore; breath offensive; ulcers healing rapidly. Omittatur calomel.

21st. Ulcers quite healed.

22d. Dressings, &c. discontinued: his mouth continued sore for some days longer, when he returned to his duty in good health.

H. M. S. Royal Sovereign, Plymouth Sound.

May 3d, 1815. Richard Lodge, ætat. 25, Ord. complained of acute pains in his bones generally, especially aggravated by the warmth of his hammock at night. Upon inspecting his body, his arms, thighs and legs, were covered with a scabby eruption, encircled by a copper-coloured base. Upon further inquiry, he informed me, that some months previous, he had had a venereal complaint, of which (to use his own phrase) he had not been properly cured. Appetite and bowels pretty natural. Three grains of opium were administered immediately; and three hours afterwards, submur. hydrarg. ℈j, which only excited a little nausea, and very little purging.

4th. *Mercurial fœtor was very evident in his breath;* and to keep up the mercurial stimulus, he took five grains of the blue pill twice a-day.

8th. Opium and calomel repeated, with the same effect.

9th. Blue pill continued; mouth becoming very sore, with rather profuse ptyalism. From this period to the 25th, his mouth continued very sore, with ptyalism for some time; the eruption dying away rapidly, and his nocturnal pains had left him. Some blue pill was continued to keep up the irritation, and by the 8th of June he returned to his duty perfectly cured.

I shall add two cases more, extracted from the Rochefort's Journal, of many more equally conclusive; they were secondary affections, and therefore afford more convincing proof of the advantage of the mode of cure.

H. M. S. Rochfort, Portsmouth Harbour.

April 2d, 1816. Thomas M'Carthy, aged about 40, seaman (having entered for the ship a few days previous), complained of a copper-coloured eruption upon the hairy scalp, chest, arms, and legs: (it is worthy of notice, that in this case there was none upon his thighs, and I have remarked in many instances the partiality of syphilitic eruptions. A case was lately under my care, in which the head, arms, and body were covered as thickly as possible, and not a single pustule beyond the ilia. In the case above, on board the Sovereign, the body was exempted. I have met with the circumstance so often, that it rather strengthens my opinion in doubtful cases of such eruptions) that on the arms especially was very close, and mostly ran into each other; and there were lumpy swellings likewise on different parts of his head. He complained also of acute pain in his head, particularly severe at night, which drove him, at times, almost to distraction; and likewise of agonizing pains in his shin bones, especially aggravated by the warmth of his bed. The eruption, he said, had not appeared above three or four days. He informed me, that about five months previous, he had a swelling in his groin, which was dispersed by the use of cold applications, without the aid of mercury; the surgeon to whom he applied having assured him that it originated solely from cold, and not from any venereal taint, to which he himself was inclined to attribute it. (Here, by the way, I cannot avoid making the following remark: Twenty years pretty attentive observation, and not a very limited experience, authorize me to observe, that, however it may generally fare with those on shore, who incur the risk of venereal contamination, with sea-faring men there is but one nearly uniform result. Let the defoedation of the object of their amour be what it may, they are little concerned. Impelled by strong and heedless desire, they rush into gratification, and *syphilis vera,* without alloy, in by far the greatest number of instances, is the *sure* reward of their temerity! The vast number of secondary affections that have come under my notice, from the primary disorder being either negligently treated, or considered to be *not venereal,* convinced me, that navy surgeons cannot be too careful in their prognosis upon these affections). His functions otherwise seemed in a pretty natural state, only he appeared much emaciated, probably from starvation on shore; he being one, of several others, labouring under similar symptoms, who seemed to have entered at this period merely to get themselves cured; and whom, from the want of better hands, we were forced to accept. (This person's life, with several others, I am convinced, would soon have been in imminent danger, had their disease not been speedily counteracted by scruple doses of calomel). He took a cathartic in the morning, about six o'clock, P. M. three grains of opium, and two hours afterwards a scruple of calomel Twelve hours afterwards, he had had only one stool; he found himself extremely drowsy, and complained of a sharp pain in his head. In the evening he had evidently the mercurial *fœtor in his breath,* though he had been *purged ten times* in the course of the day.

4th. Had exceedingly severe pain in his head last night; mouth slightly sore; no purging in the night. *Vesp.* Opium and calomel, as before.

5th. Was not purged till four o'clock this morning, since which he has had three loose motions; does not complain of much head-ache to day; mouth getting very sore.

6th. Mouth this morning (to use his own expression) is "*cruel sore,*" and the eruption has already begun evidently to diminish; has been griped and purged a good deal this morning. No medicine.

7th. Has already no pain except in his mouth, which is very sore, and he now enjoys undisturbed sleep; whereas, before he took the calomel, he was deprived of it, and suffered the most excruciating nocturnal pains.

8th. Eruption disappearing fast, mouth very sore; ptyalism considerable.

14th. Since last report his mouth has continued exceedingly sore. There is now scarcely a vestige of the eruption remaining on any part of his body, and the tumefactions on his head are entirely gone, though *two scruples only* of the submuriate have been exhibited.

16th. Excepting the soreness of his mouth, he is nearly free from complaint. In consequence of the approximation of the sick berth to a prison for captured smugglers, he and others, similarly affected, must be sent to Haslar Hospital.

John Jackson, Ab. by birth a Dane, aged about 38, complained, on the evening of the 4th of October 1817, of a pretty extensive (at least three inches in length) firm swelling on his left shin about its middle, which was extremely painful at night, but he had but little exemption even in the day. A little while previous, he had been relieved from one of the ship's tenders, from his labouring under a rheumatic affection; and this tumefaction on the shin, it appeared, had not been noticed before that illness. He positively declared, that he had not suffered any venereal affection for the last four years, and that the last disorder he had of that nature was a gonorrhœa. He took a cathartic, and was directed to use mercurial friction on the part twice a-day; and he took likewise ten grains of the blue pill in twenty-four hours. The system by these means became impregnated; the mercurial action was kept up for a month, when he had obtained so much relief, and the swelling appeared so much diminished, that he returned to his duty.

About ten days after his discharge, he again complained of his nocturnal pains having returned as bad as ever, and that the node had increased. Particular inquiry was made again into the exact state of his digestive functions, and his appetite, alvine evacuations, and other excretions appeared perfectly natural; therefore, unless the affection might be considered a sequela to his late rheumatic affection, and, perhaps, of a gouty nature, I could not consider it in any other light, than as a syphilitic disorder; especially as I felt little disposed to attend to the, too frequently, idle tales of sailors respecting their amours. In order to satisfy myself, therefore, I ordered him three grains of opium in the evening, and an hour afterwards a scruple of calomel. Following morning, (November 13th) I found that he had spent a restless night, and had been much griped and purged. Haust. anodyn. h. s. 14th. Says, that he has been purged since yesterday morning, at least, twenty times; belly still relaxed, and he finds himself weak in consequence. Opium and calomel repeated in the evening. 15th. Has been a good deal purged, but not so violently as before; is already under the influence of the mercury, and the pain of the node is greatly diminished, and he fancies it reduced in size. An opiate at night. 16th. Slightly purged; mouth sore; appetite bad. Two o'clock, P. M. Sumat opii gr. ij; hora sexta, gr. iij; et hora octo, hydrarg. submur. ℈j. 17th. Slept remarkably well last night, and was not purged till eight o'clock this morning, since which he has had three loose motions. 19th. Has been frequently purged since yesterday morning, and the soporific effect of the opium has not yet subsided. 20th. The swelling has almost disappeared; so rapid has the process of absorption been. No pain; mouth very sore, and he spits a good deal. 24th. Let the opium and calomel be repeated in the evening, as formerly; et cras mane hora 5, sumat opii gr. ij. 25th. Slept badly; was twice purged in the night; is still considerably under the influence of the narcotic; complains of sickness at stomach; mouth very sore; *swelling entirely gone.* 26th. Drowsiness continues; sligthly griped; no complaint, except soreness of his mouth. On the 1st of December he repeated the opium and calomel; his mouth afterwards continued sore until the 8th; and on the 10th, he returned to his duty; his general health, and appearance of his countenance being very much improved.

I feel it my duty to state, that about the 10th of January 1818, he again complained that the swelling was beginning to return, and that he had, for some nights previous, suffered a good deal of pain. His bowels were again emptied, and he was put upon the mercurial regimen in another form, viz. the solution of the oxymuriate in the proportion of about two grains dissolved in two ounces of rectified spirits and six of water. He commenced with an ounce thrice a-day, and increased it as his stomach could bear. In three weeks time the node again entirely disappeared.

The foregoing, which are a most faithful transcript from the original cases, I hope, will be deemed quite sufficient, to establish a striking testimony of the rapidity with which calomel administered in scruple doses, impregnates the system, and, consequently, subverts the syphilitic virus. They exhibit too, that the remedy may be employed in that proportion with perfect safety to the patient; for, from many hundreds of such doses, which I have been constantly in the habit of exhibiting since 1806, in the climate of this country, and in all seasons; not only for the cure of syphilis, but, with admirable benefit, in febrile, and other affections of the chronic description, as dysentery, hepatitis, &c. I have never observed, *in a single instance,* any alarming, or even untoward effect to proceed from them. Experiments which I made on my own person in India, in 1804, when in perfect health, prove how little excitement such doses give to the alimentary canal, in that climate. After swallowing three scruples in twelve hours, I suffered no uneasiness in my bowels whatever; on the contrary, I experienced a remarkably soothing sensation along the whole course of the canal, barely followed by a loose motion twelve hours afterwards,—but, with the impregnation of the system, which did not subside for many days. Emboldened by such a result in my own case, I administered it in the same manner, in some hundreds of instances afterwards, for the cure of dysentery, with the certain effect of immediately impregnating the system, alleviating every symptom, and speedily curing the disease, in almost every instance; as my journals of the Sceptre and Weymouth, lodged at the Transport Office, afford ample testimony. I am warranted too, from experience, in observing, that in this country, in chronic cases of dysentery and hepatitis, if medicine can be available, scruple doses of calomel itself, or in conjunction with opium, will be found the remedy, of all others, perhaps, the most to be depended upon. Twelve years experience, also, in a number of instances, induced me to think, that *confirmed syphilis*, may be *radically* cured, in a much shorter period, and with much greater safety to the constitution of the patient, than by the usual modes of employing mercury; for by its attacking the poison so instantaneously, the strength of the patient is preserved, which, from protracted courses of mercurial remedies, so frequently necessary in confirmed syphilitic affections, has, very often, declined into a state of debility, very difficult to be counteracted, and in many instances, terminating in the patient's destruction, as every one must have observed. It is highly probable too, that the opium combined with the calomel (as in the cases above stated) may have a happy tendency, in supporting the energy of the constitution; but as that is an inquiry, at present foreign to my purpose, I leave it to the judgement of others to decide.

It is probable I may be asked—Why employ calomel in such large proportions, when the virus can be effectually eradicated, by giving it in much smaller doses, or by employing other more harmless forms of mercury? To which I reply, that the disease may be unquestionably, and daily is, cured by exhibiting the remedy in smaller proportions; but, I must also affirm, that I never knew it so speedily cured, nor, in confirmed cases, perhaps, so effectually, as by giving the medicine in such doses; for I can find only two instances in my notes, of a relapse after employing scruple doses. One occurred in the Gibraltar, when two scruple doses removed a chancre; and supposing the cure perfected, no more was administered. About four months afterwards, however, secondary symptoms, in the form of eruption, appeared, which were speedily, and radically, cured by a similar administration of the medicine, oftener repeated. Another happened in the Rochfort. Previous to the battle of Algiers, the patient had taken a few scruples for the cure of chancres which had disappeared. While yet on the sick-list, he was drafted, as a volunteer, into the Queen Charlotte; and after the return of that ship to Portsmouth, and the respective drafts were sent back to their ships, I found him labouring under secondary symptoms, for which he was sent to Haslar Hospital for cure.

Another objection possibly may be made—that catharsis, even to an immoderate degree, and distressing sickness at the stomach, and griping, frequently result from scruple doses, when other more usual methods of treatment, produce little, or none of these effects? My answer to this objection would be, that so long as the soreness of the mouth. and excitement of the salivary organs, announce the impregnation of the system, the excitement of the other excretories of the body, perhaps, accelerates the cure; for if the excretories of the salivary system are simultaneously excited with those of the alimentary canal, those that open into the other internal cavities of the body, as well as those which open on the external surface, participate in the same increased action, (hence, perhaps, the peculiar fœtor of the breath; the increased sensibility to cold); whereby the inherent poison is more immediately, and, in my opinion, more certainly expelled. Analagous, at any rate, seem to have been the views of the illustrious Cullen. (Mat. Med. Vol. II. p. 450.) "This mark of mercury's stimulating the whole system, with what was said above of its affecting the whole excretories, will sufficiently show, that in its ordinary operation, by its promoting *all the excretions*, it may, therefore, *evacuate every poison*, that shall happen to be present in the mass of blood, *and thereby entirely cure the venereal disease.*"

JAMES CUNNINGHAM, Surgeon.

His Majesty's Ship Rochefort.

